# Digital Inclusion for Neurodiverse and Vulnerable Communities in the Global South: A Policy Analysis Study

**DOI:** 10.1177/00469580261443129

**Published:** 2026-04-24

**Authors:** Ahmed Yahya Almakrob, Ahmed Alduais, Alex Mhone, Borey Be, Tahira Yasmeen, Tariq Alanazi

**Affiliations:** 1 Department of English Language and Literature, College of Sciences and Humanities, Prince Sattam bin Abdulaziz University, Al-Kharj, Saudi Arabia; 2 Department of Psychology, Norwegian University of Science and Technology, Trondheim, Norway; 3 Department of Geography and Social Studies, Faculty of Education, Catholic University of Malawi, Lilongwe, Malawi; 4 Cooperation Committee for Cambodi, Phnom Penh, Cambodia; 5 School of Education, Minhaj University Lahore, Pakistan

**Keywords:** digital inclusion, neurodiversity, neurodiverse populations, good health and well-being, quality education, reduced inequalities, framework analysis

## Abstract

**Introduction:**

Digital inclusion, understood as a multidimensional construct extending beyond basic access to encompass skills, support, and meaningful participation, remains inadequately conceptualized in national policy frameworks, particularly for neurodiverse populations in Global South states such as Saudi Arabia.

**Methods:**

A qualitative policy analysis was conducted using Framework Analysis, examining 9 policy and grey documents from Saudi Arabian governmental bodies, including accessibility policies, disability legislation, digital government guidelines, and strategic frameworks. Documents were analyzed through systematic familiarization, thematic framework development, indexing, charting, and interpretation stages. The analysis was aligned with Sustainable Development Goals 3, 4, and 10.

**Results:**

Ten thematic areas were identified. Digital inclusion efforts rely primarily on international accessibility benchmarks and technical compliance standards, with underlying assumptions frequently conflating disability with digital illiteracy. While the 2019 Disability Law and Vision 2030 represent significant policy advancements, inconsistencies persist in support definitions and implementation across sectors. Reported outcomes include improved health service delivery—such as sign language interpretation services reaching over 35 000 individuals—and educational platform success, notably the Madrasati platform recognized globally during COVID-19. Persistent barriers include physical inaccessibility, digital literacy deficits, sensory and cognitive constraints, and deficit-oriented policy language.

**Conclusion:**

Achieving meaningful digital inclusion requires shifting from technical conformity to user-centered design, establishing consistent vulnerability definitions, investing in capacity-building, and implementing SDG-aligned monitoring systems to track progress and support adaptive policy refinement for vulnerable populations.

## Introduction

### Conceptual Foundations of Digital Inclusion

Digital inclusion is widely understood as a comprehensive, multidimensional construct that extends beyond basic access to digital tools, encompassing the skills, support, and opportunities individuals need to participate meaningfully in a digital society. Scholars emphasize that digital inclusion functions as the counterpart to digital exclusion, involving not only the provision of robust broadband and appropriate devices but also the development of digital literacy, ongoing technical support, and access to content that enables participation, collaboration, and self-sufficiency structures that address diverse user needs, particularly in rapidly digitizing environments.^1-5^ Central definitions highlight that the transition from exclusion to inclusion requires achieving both parity in ICT access and equality in digital skills, usage, and participation structures that address diverse user needs, particularly in rapidly digitizing environments.^4-7^ Consequently, digital inclusion refers not simply to the availability of infrastructure but to an ecosystem of interconnected factors that allows individuals and communities to fully benefit from technological and societal advancements. This perspective underscores the importance of integrating inclusive design, user-centered content, and support structures that address diverse user needs, particularly in rapidly digitizing environments.^4,5^

The literature further positions digital inclusion within broader frameworks of social inclusion, noting that digital inequalities often mirror and reinforce pre-existing social, economic, and demographic disparities. While the digital divide focuses primarily on disparities in access, digital inclusion encompasses the sociocultural and structural barriers that shape individuals’ ability to leverage digital technologies, including illiteracy, language differences, geographic isolation, and age-related or disability-related challenges.^2,8,9^ Research also emphasizes the intersectional nature of digital disadvantage, whereby factors such as socioeconomic status, disability, and marginalization can cumulatively compound barriers to inclusion.^4,10^ In response, policy and community initiatives increasingly focus on ensuring equitable participation by tailoring digital literacy programs, providing alternative content formats, and designing interventions that meet the needs of vulnerable groups such as older adults, people with disabilities, and those with low-income backgrounds.^11,12^ As digital environments evolve, scholars argue that digital inclusion must be understood as a dynamic process requiring continual adaptation, as new technologies and platforms create new forms of exclusion and reshape what it means to participate fully in digital life.^13-15^

### Policies and Initiatives Supporting Digital Inclusion for Neurodiverse and Vulnerable Groups

International research highlights a rapidly expanding landscape of policies and initiatives aimed at supporting digital inclusivity for neurodiverse individuals and vulnerable populations. Disability-specific policies consistently demonstrate greater effectiveness than broad-based diversity frameworks because they explicitly acknowledge the needs, rights, and unique strengths of neurodivergent individuals, thereby reducing bias, improving workplace climate, and enhancing awareness of available accommodations.^16^ Organizational initiatives within healthcare, education, and employment contexts increasingly prioritize tailored training, reasonable accommodations, and inclusive leadership practices to cultivate safer and more supportive environments for neurodivergent people.^17^ National legislative frameworks further reinforce digital inclusion, such as the US Developmental Disabilities Act and Rehabilitation Act, Brazil’s legal recognition of autism as a disability, and targeted educational policies in England, Scotland, and Ireland, all of which mandate accessible learning opportunities and social benefits.^18^ Broader digital inclusion policies, including Hungary’s National Digitalization Strategy and similar international programs, focus on infrastructure, digital skills, and e-health initiatives for socially marginalized groups, although researchers note that device-distribution alone is insufficient without deeper structural and culturally responsive support mechanisms.^19^ Collectively, these policies illustrate a global trend toward embedding accessibility, inclusion, and legal protection within digital governance frameworks to better support individuals who face systemic barriers.

In parallel with legislative efforts, policy shifts increasingly incorporate inclusive design principles, co-design methodologies, and emergent technologies to meaningfully engage neurodiverse communities in digital transformation. Scholars emphasize the need for mandatory disability co-design requirements in digital government projects, arguing that authentic collaboration with people with disabilities leads to more equitable, innovative, and accessible public digital services interfaces to personalized feedback and multimodal interaction—are increasingly used to support neurodivergent users across healthcare, employment, and education settings, though research highlights persistent gaps in user participation, long-term evaluation, and ethical safeguards.^20^ Emerging digital environments, such as the Metaverse, present new opportunities for accessibility through universal design, assistive technology integration, and global standards, but also raise concerns related to interoperability, equity, and privacy.^21,22^ Policy scholars further caution that digital inclusion initiatives can inadvertently reinforce stereotypes if they over-target certain groups without adopting universal, stigma-free strategies.^23^ Ethical governance frameworks stress the importance of embedding fairness, inclusiveness, and human rights principles throughout the lifecycle of digital systems, particularly for populations historically excluded from technological innovation.^24^ Together, these developments underscore that digital inclusion for neurodiverse and vulnerable individuals requires not only technical accessibility but also policy coherence, participatory design, ethical safeguards, and sustained investment in digital literacy and community support.^25^ Cultural and educational institutions are also adopting inclusive design practices, reframing engagement through neurodiverse perspectives to dismantle cognitive, sensory, and digital barriers interfaces to personalized feedback and multimodal interaction—are increasingly used to support neurodivergent users across healthcare, employment, and education settings, though research highlights persistent gaps in user participation, long-term evaluation, and ethical safeguards.^20^ Emerging digital environments, such as the Metaverse, present new opportunities for accessibility through universal design, assistive technology integration, and global standards, but also raise concerns related to interoperability, equity, and privacy.^21,22^ Policy scholars further caution that digital inclusion initiatives can inadvertently reinforce stereotypes if they over-target certain groups without adopting universal, stigma-free strategies.^23^ Ethical governance frameworks stress the importance of embedding fairness, inclusiveness, and human rights principles throughout the lifecycle of digital systems, particularly for populations historically excluded from technological innovation.^24^

Together, these developments underscore that digital inclusion for neurodiverse and vulnerable individuals requires not only technical accessibility but also policy coherence, participatory design, ethical safeguards, and sustained investment in digital literacy and community support.^26^ Technology-driven initiatives—ranging from AI-powered adaptive interfaces to personalized feedback and multimodal interaction—are increasingly used to support neurodivergent users across healthcare, employment, and education settings, though research highlights persistent gaps in user participation, long-term evaluation, and ethical safeguards.^20^ Emerging digital environments, such as the Metaverse, present new opportunities for accessibility through universal design, assistive technology integration, and global standards, but also raise concerns related to interoperability, equity, and privacy.^21,22^ Policy scholars further caution that digital inclusion initiatives can inadvertently reinforce stereotypes if they over-target certain groups without adopting universal, stigma-free strategies.^23^ Ethical governance frameworks stress the importance of embedding fairness, inclusiveness, and human rights principles throughout the lifecycle of digital systems, particularly for populations historically excluded from technological innovation.^24^ Together, these developments underscore that digital inclusion for neurodiverse and vulnerable individuals requires not only technical accessibility but also policy coherence, participatory design, ethical safeguards, and sustained investment in digital literacy and community support.

### Digital Inclusion for People With Disabilities in Saudi Arabia

Digital inclusivity for people with disabilities in Saudi Arabia is advancing under a strong national policy and legal framework shaped by the Law of Disability and Vision 2030s transformative agenda for health and education services. Legislative reforms mandate equal access to medical, psychological, social, and educational services for individuals with disabilities, with more recent updates emphasizing accessibility, vocational services, and accommodations across all educational stages.^27,28^ In healthcare, digital transformation initiatives are increasingly driven by advanced technologies including artificial intelligence, machine learning, Internet of Things solutions, and telemedicine platforms designed to enhance accessibility and autonomy.^29,30^ AI-powered tools such as federated learning models, zero-shot architectures, and personalized chatbots have been proposed to address long-standing challenges in data processing, diagnostic delays, and accessibility—particularly for visually impaired users.^29^ These technological expansions offer promising means for remote healthcare delivery, real-time monitoring, and personalized intervention planning.^31^ However, their impact remains constrained by persistent barriers including infrastructure gaps in rural regions, connectivity issues, usability challenges, and limited digital literacy and training among both practitioners and service users.^32,33^ Provider-level evidence shows that less than half of healthcare practitioners regularly use telemedicine with individuals with disabilities, and usability outcomes remain moderate, largely driven by practitioner competency and satisfaction with available telehealth resources.^30^ These findings demonstrate that while the technological foundation for inclusive digital healthcare is expanding, continued progress depends on strengthening infrastructure, enhancing user-centered design, and scaling workforce training.

In education, Saudi Arabia has made notable advances through policies that mandate inclusive schooling and guarantee equal access to meaningful, supportive learning opportunities for students with disabilities, including those with visual, intellectual, and sensory impairments).^35^ Digital transformation efforts show varied impact across disability groups: families of individuals with physical and hearing disabilities express greater optimism regarding digital education, while those of individuals with visual and intellectual disabilities report more neutral perceptions, reflecting ongoing accessibility and design limitations.^34^ At the higher education level, significant inequities persist, particularly in medical and professional training programs where admission decisions often hinge on narrow interpretations of functional capacity and insufficient accommodations.^35^ Proposed frameworks call for more inclusive admissions policies, flexible curricula, and digitally accessible learning environments that align with national priorities and sociocultural contexts. Together, these patterns demonstrate that while Saudi Arabia’s policy landscape strongly supports digital inclusivity, realizing full inclusion requires sustained investment in educator training, universal design practices, coordinated institutional support, and culturally responsive technologies tailored to the needs of diverse learners.^27,35,36^ Digital transformation efforts show varied impact across disability groups: families of individuals with physical and hearing disabilities express greater optimism regarding digital education, while those of individuals with visual and intellectual disabilities report more neutral perceptions, reflecting ongoing accessibility, and design limitations.^34^ At the higher education level, significant inequities persist, particularly in medical and professional training programs where admission decisions often hinge on narrow interpretations of functional capacity and insufficient accommodations.^35^ Proposed frameworks call for more inclusive admissions policies, flexible curricula, and digitally accessible learning environments that align with national priorities and sociocultural contexts.

Together, these patterns demonstrate that while Saudi Arabia’s policy landscape strongly supports digital inclusivity, realizing full inclusion requires sustained investment in educator training, universal design practices, coordinated institutional support, and culturally responsive technologies tailored to the needs of diverse learners. Digital transformation efforts show varied impact across disability groups: families of individuals with physical and hearing disabilities express greater optimism regarding digital education, while those of individuals with visual and intellectual disabilities report more neutral perceptions, reflecting ongoing accessibility, and design limitations.^34^ At the higher education level, significant inequities persist, particularly in medical and professional training programs where admission decisions often hinge on narrow interpretations of functional capacity and insufficient accommodations.^35^ Proposed frameworks call for more inclusive admissions policies, flexible curricula, and digitally accessible learning environments that align with national priorities and sociocultural contexts. Together, these patterns demonstrate that while Saudi Arabia’s policy landscape strongly supports digital inclusivity, realizing full inclusion requires sustained investment in educator training, universal design practices, coordinated institutional support, and culturally responsive technologies tailored to the needs of diverse learners. reinforces these guarantees by requiring accessible curricula, assistive technologies, individualized strategies, transition programs, and vocational preparation across the educational lifespan 2024). Digital transformation efforts show varied impact across disability groups: families of individuals with physical and hearing disabilities express greater optimism regarding digital education, while those of individuals with visual and intellectual disabilities report more neutral perceptions, reflecting ongoing accessibility, and design limitations.^34^ At the higher education level, significant inequities persist, particularly in medical and professional training programs where admission decisions often hinge on narrow interpretations of functional capacity and insufficient accommodations.^35^ Proposed frameworks call for more inclusive admissions policies, flexible curricula, and digitally accessible learning environments that align with national priorities and sociocultural contexts.

All in all, these patterns demonstrate that while Saudi Arabia’s policy landscape strongly supports digital inclusivity, realizing full inclusion requires sustained investment in educator training, universal design practices, coordinated institutional support, and culturally responsive technologies tailored to the needs of diverse learners.^37^ Despite these commitments, implementation challenges persist. Studies highlight gaps in specialized teacher training, limited capacity to meet diverse learning needs, curricular rigidity, and insufficient parental engagement, all of which hinder equitable participation.^38,39^ Digital transformation efforts show varied impact across disability groups: families of individuals with physical and hearing disabilities express greater optimism regarding digital education, while those of individuals with visual and intellectual disabilities report more neutral perceptions, reflecting ongoing accessibility, and design limitations.^34^ At the higher education level, significant inequities persist, particularly in medical and professional training programs where admission decisions often hinge on narrow interpretations of functional capacity and insufficient accommodations.^35^ Proposed frameworks call for more inclusive admissions policies, flexible curricula, and digitally accessible learning environments that align with national priorities and sociocultural contexts. Together, these patterns demonstrate that while Saudi Arabia’s policy landscape strongly supports digital inclusivity, realizing full inclusion requires sustained investment in educator training, universal design practices, coordinated institutional support, and culturally responsive technologies tailored to the needs of diverse learners.

Although growing policy commitments, neurodiverse and vulnerable populations in Saudi Arabia remain underserved in terms of digital access. The prevalence of disability has risen from 0.8% in 2004 to 7.1% in 2017,^40,41^ with neurodiverse conditions such as ADHD and autism spectrum disorder being the most studied, yet still poorly understood due to persistent knowledge gaps and stigma.^42^ Neurodiversity practices in education—including teacher preparedness, assistive technologies, and inclusive infrastructure—remain underdeveloped,^43^ while the majority of individuals with severe mental health conditions do not seek treatment.^42^ These patterns reflect broader digital exclusion dynamics: low digital literacy, limited accessible services, and fragmented support across health and educational systems.^44,45^

In short, significant gaps remain in the existing literature albeit policy advances are realized. First, knowledge about neurodiverse and vulnerable populations in Saudi Arabia—including their digital access experiences, support needs, and engagement with health and educational technologies—remains fragmented and undertheorised.^42,43^ Second, while international scholarship has advanced frameworks for digital inclusion, these have rarely been applied to systematically analyze national policy documents from Global South contexts in relation to specific SDG targets. Third, the dual domains of digital health and digital education are typically studied in isolation, obscuring the cross-sectoral gaps that most affect people with disabilities and neurodevelopmental differences. The present study addresses these gaps by conducting a structured policy analysis that examines how national documents conceptualize, evidence, and operationalize digital inclusion for neurodiverse and vulnerable populations across both domains.

### The Present Study

In Saudi Arabia and the wider Global South, healthcare and education often operate as separate systems, resulting in fragmented services and limited continuity of support for people with disabilities or neurodevelopmental differences. This separation contributes to gaps in accessibility, uneven adoption of assistive technologies, and low integration between digital health and digital learning environments, as noted in recent work highlighting systemic barriers and limited cross-sector collaboration priorities. To guide this analysis, 9 policy and grey documents were examined with reference to Sustainable Development Goals (SDGs) central to digital inclusion: SDG 3 (Good Health and Well-being), SDG 4 (Quality Education), and SDG 10 (Reduced Inequalities), along with Target 3.8 on universal health coverage, Target 4.5 on equitable access to education, and Indicators 3.8.1 and 4.5.1. These were selected for their relevance to accessibility, equity, and participation in digital health and education. The study is guided by 3 aims: (1) to examine how digital inclusion for neurodiverse and vulnerable populations is conceptualized and framed in national policy documents; (2) to identify the evidence, outcomes, and challenges described in relation to digital access in health and education; and (3) to analyze the actions, stakeholders, and strategies proposed to advance equitable digital participation for these populations.^43,46^ The present study responds to these gaps by examining how national policy documents describe and support digital participation across both health and education, and by exploring how digital technologies might serve as a shared ecosystem to strengthen communication, continuity of care, and inclusive learning. Building on the earlier Saudi context, the study focuses on how policy texts articulate rights, responsibilities, and support mechanisms for neurodiverse and vulnerable groups, particularly in relation to national priorities. To guide this analysis, 9 policy and grey documents were examined with reference to Sustainable Development Goals (SDGs) central to digital inclusion: SDG 3 (Good Health and Well-being), SDG 4 (Quality Education), and SDG 10 (Reduced Inequalities), along with Target 3.8 on universal health coverage, Target 4.5 on equitable access to education, and Indicators 3.8.1 and 4.5.1. These were selected for their relevance to accessibility, equity, and participation in digital health and education. The study is guided by 3 aims: (1) to examine how digital inclusion for neurodiverse and vulnerable populations is conceptualized and framed in national policy documents; (2) to identify the evidence, outcomes, and challenges described in relation to digital access in health and education; and (3) to analyze the actions, stakeholders, and strategies proposed to advance equitable digital participation for these populations.

## Methods

### Design

The study adopted a qualitative policy analysis design using Framework Analysis, a structured methodological approach well suited for examining complex policy environments and systematically comparing thematic patterns across multiple documents.^47^ Framework Analysis was selected because it allows researchers to integrate both inductive and deductive perspectives while maintaining transparency and rigor in how interpretive decisions are made. This design is especially appropriate for studies addressing health, education, and social policy, where clearly defined stages such as familiarization, framework development, indexing, charting, and mapping support a coherent analytic process.^48^ In this study, the design centered on analyzing 9 policy and grey documents addressing digital inclusion for neurodiverse and vulnerable populations in Saudi Arabia, including national accessibility policies, disability legislation, digital government guidelines, and initiatives from key governmental bodies. These materials were examined to understand how digital inclusion is conceptualized, the extent to which vulnerable groups are explicitly addressed, and how national strategies align with international accessibility standards and global equity objectives. The design enabled a structured comparison of definitions, assumptions, barriers, recommendations, and stakeholder roles across documents, ensuring an in-depth and systematic synthesis of policy commitments and gaps relevant to the Saudi digital inclusion landscape. Document collection and analysis were conducted between July and October 2025. Finally, it should be noted that this paper was prepared and edited in accordance with the Standards for Reporting Qualitative Research (SRQR) reporting guideline,^49^ and the completed checklist is provided as a Supplemental File.

### Sample

The sample consisted of 9 policy and grey documents selected through purposive sampling to ensure that only materials directly relevant to digital inclusion in Saudi Arabia were included. This approach was appropriate because the study required documents that explicitly address national strategies, regulatory frameworks, accessibility standards, and initiatives related to the inclusion of neurodiverse individuals and other vulnerable populations in digital health and education systems. The sampled documents represent a diverse set of governmental and semi-governmental sources, including accessibility policies, national disability legislation, digital government guidelines, strategic frameworks, and programmatic reports. Together, they provide a comprehensive overview of current national efforts, regulatory commitments, and operational practices shaping digital inclusion in Saudi Arabia. Each document contributed unique insights into definitions of accessibility, implementation mechanisms, barriers, and institutional responsibilities relevant to the study’s analytic objectives.

All 9 documents are freely accessible online via the official websites of the respective Saudi governmental bodies as detailed in Table 1. They were retrieved through systematic searches of institutional portals, including those of the Ministry of Human Resources and Social Development, the Digital Government Authority, the Authority of People with Disability, and the Saudi Data and Artificial Intelligence Authority. Full URLs are provided in the reference list, where documents are marked with an asterisk.

**Table 1.** Policy and Grey Documents Included in the Sample.

Document title	Issuing authority
1. Accessibility Policy	Ministry of Human Resources and Social Development
2. Disability Law (2019) Overview	Skynet Technologies (reporting on national legislation)
3. Digital Government Policies (v2.0)	Digital Government Authority
4. Digital Inclusion National Platform	Digital Government Authority
5. Introductory Guide: Digital Inclusion Program	Digital Government Authority
6. Overview of Digital Inclusivity in Digital Government	Digital Government Authority
7. Overview of the Rights of Persons with Disabilities	Authority of People with Disability
8. Digital Inclusion Strategy	Saudi Data and Artificial Intelligence Authority
9. Disability Empowerment Report	Saudi Data and Artificial Intelligence Authority

Documents were included if they: (1) were produced or officially endorsed by a Saudi Arabian governmental or semi-governmental body; (2) explicitly addressed digital inclusion, digital accessibility, disability rights, or digital government policy in the Saudi national context; and (3) were publicly available in full text. Documents were excluded if they: (1) were produced by non-governmental or international bodies without direct relevance to the Saudi national policy context; (2) addressed digital inclusion in other national contexts without reference to Saudi Arabia; or (3) were not available in full text for systematic analysis. Grey literature items were included where they directly reported on official Saudi national programs or legislative frameworks, as confirmed by cross-referencing with primary governmental sources. Each document was carefully reviewed to ensure a clear understanding of the context and content it provided. This foundational step involved familiarizing the researcher with the overarching themes, specific terminology, and regulatory frameworks referenced in each document.

### Measures

To ensure the trustworthiness and credibility of the analysis, the study incorporated established qualitative research strategies aligned with best practices for document-based policy analysis. Credibility was strengthened through prolonged engagement with the 9 policy and grey documents, allowing for repeated readings and sustained interaction with the material to deepen understanding of contextual nuances, terminology, and policy intentions. Triangulation was achieved by examining documents originating from multiple governmental bodies and agencies, including accessibility policies, disability legislation, digital government frameworks, and strategic reports. Using varied document types enhances credibility by enabling the researcher to cross-check consistencies and discrepancies across sources.^50^

Dependability was supported through the systematic use of Framework Analysis, which provides a transparent and replicable analytic structure. By following clearly defined stages such as familiarization, framework development, coding, matrix charting, and interpretation, the study ensured that analytic decisions were traceable and grounded in the data.^48^ To reinforce confirmability, an audit-like trail was maintained through documentation of coding decisions, analytic memos, and the rationale for thematic grouping. This reduces researcher bias by making the analytic process explicit and open to scrutiny.^51^

Transferability was enhanced by providing detailed descriptions of the documents analyzed and the context in which Saudi digital inclusion policies operate. The inclusion of diverse policy sources allows readers to assess the applicability of findings to other national or sectoral contexts. Additionally, reflexivity was maintained throughout the analysis, acknowledging the researcher’s positionality, professional background, and potential influence on interpretation. Together, these strategies collectively contribute to a rigorous and trustworthy assessment of how digital inclusion is conceptualized, operationalized, and aligned with broader equity and accessibility goals across the Saudi policy landscape.

### Procedure

This analysis utilized a Framework Analysis methodology as described by Ritchie and Spencer,^47^ specifically focusing on its Content and Framework approaches relevant in an academic context. The purpose of this methodology is to provide a structured way to synthesize qualitative data extracted from various documents related to digital inclusivity in Saudi Arabia. By emphasizing a high-level, stage-based approach, this analysis ensures that the complexities inherent in the material are comprehensively addressed. The methodology unfolded through several key stages: familiarization with the data, identifying a thematic framework, charting data into a matrix, and ultimately mapping and interpreting the findings.

Data extraction was performed through a meticulous process aimed at identifying key themes and insights relevant to the research questions. This involved a mix of thematic and content analyses where significant passages were highlighted, and recurring themes such as barriers, stakeholder involvement, policy outcomes, and language framing were identified. By charting these themes into a structured matrix, the analysis facilitated a clearer comparison and interpretation of how different documents align or diverge in their approach to digital inclusivity. The synthesis of findings involved not only aggregating the themes but also weaving together evidence drawn from multiple sources to create a coherent narrative that reflects the current state of digital inclusion efforts in Saudi Arabia. To ensure rigor and minimize interpretive bias, all 6 authors participated in the document review process. Each document was independently read by at least 2 authors, following which the team convened to compare observations, resolve discrepancies, and reach consensus on thematic coding and analytical decisions. This collaborative approach strengthened the credibility and confirmability of the analysis, consistent with best practices for team-based qualitative document analysis.^48,50^

In alignment with the Framework Analysis methodology developed by Ritchie and Spencer, we systematically analyzed the 9 documents concerning digital inclusion for neurodiverse and vulnerable populations, with a specific focus on their relevance to the Sustainable Development Goals (SDGs). This involved identifying key themes related to health (SDG 3), education (SDG 4), and inequality (SDG 10), as well as assessing how these documents address respective targets and indicators associated with the SDGs. Each document was examined for its explicit and implicit references to policies, initiatives, and outcomes that contribute to or reflect progress toward these global goals. By cross-referencing the content within the documents against established SDG targets and indicators, we drew inferences about the effectiveness and comprehensiveness of Saudi Arabia’s strategies for promoting digital inclusion. This integrated approach ensured that our findings remained consistent with previous analyses, highlighting the crucial connections between national policy documents and global sustainability objectives. It is important to note that in document-based policy analysis, the documents themselves constitute the data. Framework Analysis provides a structured, transparent, and auditable approach to analyzing this data, comparable in rigor to qualitative coding of interview or observational data.^48,50^ The systematic charting matrix used in this study enabled structured cross-document comparison and traceable interpretation.

## Results

**Figure 1.**
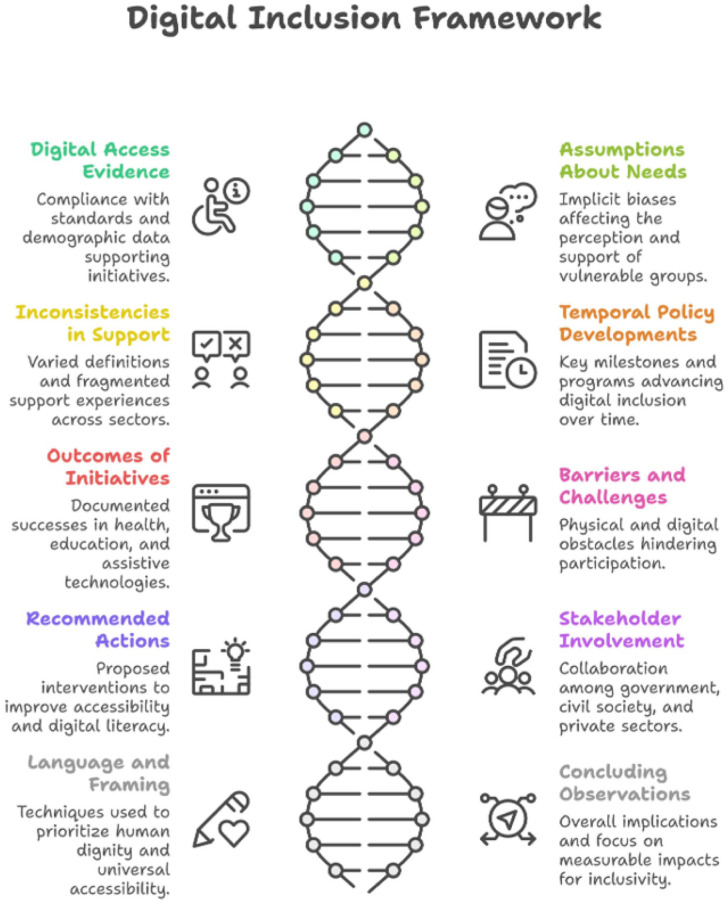
Framework of the current digital inclusion system in Saudi Arabia for persons with disabilities.

Each thematic response below is grounded in direct textual evidence extracted and charted from the 9 documents. Where cross-document patterns, contradictions, or silences are identified, these reflect systematic comparison across the analytical matrix rather than general characterization. The 10 thematic areas presented in Figure 1 were generated through the systematic application of Framework Analysis,^47,48^ specifically through the indexing and charting stages in which recurring patterns, definitions, assumptions, and policy language were identified across the 9 documents. The Q1-Q10 structure reflects the analytic questions used to guide data extraction and interpretation within this framework, ensuring that findings are grounded in the documentary evidence rather than in secondary literature.

Figure 1 synthesizes 10 major thematic areas that collectively illustrate how digital inclusivity is being framed and operationalized for vulnerable communities in Saudi Arabia. Evidence of digital access highlights the reliance on international accessibility benchmarks, demographic data, and user testing to justify targeted interventions. Underlying assumptions reveal persistent tendencies to merge disability with digital illiteracy and to prioritize technical conformity over user-centered design. The materials also show inconsistencies in support, including divergent definitions of vulnerability and uneven implementation across sectors. Policy developments—particularly the 2019 Disability Law and Vision 2030 initiatives—demonstrate a temporal progression toward more structured approaches to digital inclusion. Reported outcomes emphasize improvements in health, education, and assistive technology adoption, while barriers continue to limit participation through physical, sensory, and cognitive constraints. Recommended actions focus on standardization, digital literacy, and technological enhancement, supported by a range of stakeholders including government bodies, civil society, and individuals with disabilities. Finally, language and framing patterns influence how disability and capability are perceived, shaping the broader implications for achieving sustainable and meaningful digital inclusivity. Each of these themes is elaborated in details after the visualization.

### Q1. Which Passages Define or Describe Digital Inclusion for Neurodiverse Individuals and Vulnerable Communities in Saudi Arabia?

In examining the digital inclusion landscape in Saudi Arabia, it is important to understand how the policies and frameworks address the needs of neurodiverse individuals and vulnerable communities. The nation has enacted robust legal and regulatory measures designed to ensure equitable access to digital services for all citizens, especially those with disabilities. Central to this effort is the “Rights of Persons with Disabilities Act” required rather than optional.^52^ By codifying specific rights—like access to multimedia content and priority services for the elderly—these frameworks operationalize the commitment to digital inclusion beyond abstract notions, translating them into actionable entitlements,^53^ which mandates digital accessibility across both public and private sectors. This foundational legislation is harmonized with Vision 2030, reinforcing that compliance with international accessibility standards, such as WCAG 2.1 AAA, is required rather than optional.^52^ By codifying specific rights—like access to multimedia content and priority services for the elderly—these frameworks operationalize the commitment to digital inclusion beyond abstract notions, translating them into actionable entitlements.

The implementation of targeted assistive technologies and digital literacy initiatives reflects Saudi Arabia’s commitment to overcoming barriers faced by vulnerable populations. The Digital Inclusion National Platform emphasizes the importance of developing educational programs focused on improving digital skills among persons with disabilities and the elderly, ensuring that these groups can effectively engage with digital services.^54,55^ This is complemented by the integration of various assistive technologies, including screen readers and adaptive learning tools, which are mandated across government platforms to facilitate access and enhance user experience.^56,57^ Moreover, the introduction of digital identification systems, such as specialized cards for people with disabilities, provides privileges like transportation discounts and expedited healthcare access, illustrating a concrete commitment to making digital services more accessible and beneficial for these populations.^58^

Moreover, active participation by persons with disabilities is integral to the development and refinement of these policies. Documents clearly assert that the voices of disabled individuals—including neurodiverse users—must be central to the policy-making process for effective digital inclusion.^59^ This participatory approach is reflected in the framework established by the Digital Government Authority, which asserts the necessity of incorporating feedback from the elderly and persons with disabilities during the design phase of digital initiatives.^56^ The active involvement of affected communities ensures that the services developed are more aligned with their needs, thereby creating a more user-centered experience.

Despite these advancements, challenges remain in the specific articulation of policies catering to neurodiverse individuals. While general terms for disability cover many aspects, explicit references to neurodiversity are limited, as most documents often sort neurodiverse experiences into broader disability categories without recognizing the unique needs that different conditions demand. The terminology used, generally residual of medical models, frequently positions disability as a condition requiring accommodation rather than recognizing neurodiversity as an intrinsic aspect of human cognition requiring tailored interventions.^60^

In summary, while Saudi Arabia exhibits a strong commitment toward digital inclusion for vulnerable populations through comprehensive legal frameworks, targeted initiatives, and the active participation of affected communities, further refinement is needed. The current policies must expand to explicitly acknowledge and address the distinct experiences and needs of neurodiverse individuals to truly create an inclusive and supportive digital environment. This necessitates greater integration of specific design considerations and measurable outcomes for neurodiverse groups within the broader national strategy, ensuring that digital transformation benefits all citizens equitably.

### Q2. What Types of Evidence Support Claims About Technology Access for People With Disabilities or Other at Risk Groups?

To understand the types of evidence supporting claims about technology access for people with disabilities or other at-risk groups in Saudi Arabia, it is essential to examine the multifaceted framework established through various government documents and policies. The evidence encompasses compliance with international standards, statistical population data, user testing outcomes, and economic research that collectively illustrate the commitment to enhancing digital accessibility. Each category of evidence plays a crucial role in substantiating the claims made regarding the effectiveness of initiatives aimed at fostering inclusivity for vulnerable populations.

One of the primary forms of evidence documented across these policies is compliance with internationally recognized accessibility standards, particularly the Web Content Accessibility Guidelines (WCAG). Various documents explicitly mandate adherence to these guidelines as a foundational measure for ensuring accessibility within digital platforms. For instance, the Accessibility Policy from the Ministry of Human Resources and Social Development (2025) asserts that “all digital services adhere to the Web Content Accessibility Guidelines (WCAG) 2.1 at Level AAA” to assure accessibility for users with disabilities. Additionally, the Digital Inclusion National Platform emphasizes the requirement for all government portals to comply with WCAG 2.0 AAA standards, thereby framing technical compliance as a critical benchmark for accessibility efforts.^54^ This emphasis on international standards establishes a clear regulatory requirement that enhances accountability while facilitating traceable implementation across government entities.

Complimentary to technical compliance metrics, demographic statistics are employed within these documents to evidence the need for targeted inclusion initiatives. The Introductory Guide Digital Inclusion Program (2023) notes that “according to the latest studies, the percentage of persons with disabilities in Saudi Arabia stands at 7.1%,” reinforcing the argument for focused accessibility strategies.^56^ Similarly, the Digital Inclusion National Platform highlights that the General Authority for Statistics provides essential data to inform policy decisions regarding digital inclusion efforts. This statistical framework allows policymakers to strategize and allocate resources effectively, ensuring that initiatives are designed based on a comprehensive understanding of the population’s needs.

User testing is another significant form of evidence indicating the efficacy of digital accessibility measures. The Digital Inclusion Program highlights the importance of conducting accessibility tests and exploratory sessions with individuals with disabilities to verify the effectiveness of digital services.^56^ This practice not only validates that services function as intended for diverse user needs but also facilitates meaningful engagement with the communities served, positioning them as active participants rather than passive recipients. Such firsthand accounts and qualitative feedback contribute to a deeper understanding of actual accessibility experiences and outcomes, juxtaposing the technical specifications with lived realities.

Economic research further strengthens the rationale for investing in digital accessibility initiatives by linking technology access to broader economic benefits. The Overview of Digital Inclusion in Digital Government indicates that studies from the International Monetary Fund have found digital financial inclusion positively correlates with growth in GDP per capita.^54^ This connection transitions the discourse on accessibility from a strictly ethical consideration to a tangible business case, illustrating how improving digital access can yield substantial economic returns. By framing these initiatives as economically beneficial, policymakers can better justify their investments, supporting the argument for inclusive practices as advantageous not only for social equity but also for national development.

However, gaps remain across the evidence landscape, particularly concerning detailed reports of measurable outcomes and specific engagement metrics for distinct populations. The majority of policy documents outline initiatives and compliance standards without systematically assessing the real-world impact of these actions on the lives of individuals with disabilities.^59^ While existing data and standards provide a foundation for understanding accessibility efforts, the need for more granular measurement of success—particularly for neurodiverse users—becomes apparent. Addressing these shortcomings presents an opportunity for future policy refinement, emphasizing the necessity for comprehensive monitoring and evaluation frameworks to ensure that digital inclusion efforts truly resonate with and benefit diverse populations across Saudi Arabia.

### Q3. What Assumptions Are Implied About the Digital Needs, Abilities, or Participation of Neurodiverse Learners or Users With Disabilities?

To address the digital needs and participation of neurodiverse learners and users with disabilities, it is crucial to examine the implicit assumptions found within Saudi Arabia’s policy documents and frameworks. These documents provide a structured approach to digital inclusion; however, they often reflect underlying beliefs about the abilities and requirements of individuals within these groups. For example, there is a tendency to regard neurodiverse users predominantly through a lens of limitation, implying that their cognitive differences necessitate specialized accommodations rather than recognizing the diverse capabilities inherent in neurodiversity.

Many of the policies adopt a deficit-based model, framing neurodiverse individuals primarily in relation to their perceived limitations. For example, the Accessibility Policy emphasizes the need for content and interfaces to be “easy to understand and interact with, regardless of users’ cognitive abilities.”^52^ This language reflects an assumption that users with cognitive differences are less capable of navigating standard digital environments without significant support. While aiming to create more accessible and intuitive designs is commendable, this framing inadvertently places the responsibility on the individual to adapt to the system rather than on the system to accommodate diverse cognitive styles. In contrast, a more nuanced understanding of neurodiversity would recognize cognitive differences as a spectrum of abilities that can offer unique perspectives and contributions.

Moreover, the documents tend to conflate disability with digital illiteracy, suggesting that individuals who identify as disabled require remedial efforts to engage with digital technologies fully. The Digital Inclusion National Platform outlines plans to eradicate “digital illiteracy” among populations deemed vulnerable, such as the elderly and persons with disabilities, through specialized training programs.^54^ This assumption undermines the existing digital competencies that many individuals within these populations may already possess. It also reduces neurodiverse individuals to a stereotype of incompetence, failing to acknowledge that many may have advanced digital skills or may engage with technology differently due to their unique thought processes.

The emphasis placed on compliance with international accessibility standards, while essential, often overshadows a user-centered approach that considers the diverse needs of neurodiverse learners and users with disabilities. For instance, many documents focus on implementing technical specifications, such as ensuring color contrast and providing text alternatives, as benchmarks for accessibility.^56^ However, this focus on technical compliance can overlook important qualitative aspects of user experience and participation that are critical for fostering true inclusion. The assumption that adherence to these technical standards equates to accessibility may lead to surface-level compliance rather than genuine engagement and usability for all users.

Finally, the framing of disability as a vulnerability shared among broader marginalized groups can dilute the unique rights and identity of individuals with disabilities, including those who are neurodiverse. In various documents, categories such as “vulnerable groups” are often used interchangeably with disability, encompassing women, the elderly, and individuals in remote areas without distinction.^54^ This vulnerability-based framework risks reinforcing paternalistic views that treat individuals with disabilities as passive recipients of aid rather than active participants in shaping their digital experiences. By not explicitly recognizing the distinct rights and needs of these communities, the potential for tailored interventions that genuinely enhance access and participation is diminished.

In conclusion, while Saudi Arabia’s digital inclusion efforts demonstrate an overarching commitment to accessibility, the documents often reflect assumptions that could impede meaningful engagement for neurodiverse and disabled individuals. These include deficit-based understandings of abilities, conflation with digital illiteracy, a lack of user-centered approaches, and generalized vulnerability framing. Addressing these assumptions is essential for developing policies that truly recognize and cater to the diverse needs of all users, enabling a more equitable digital landscape.

### Q4. What Inconsistencies Appear Across Documents Regarding Support for Vulnerable or Minority Groups in Digital Health or Education?

In exploring the inconsistencies across Saudi Arabian government documents regarding support for vulnerable or minority groups in digital health or education, it becomes evident that while substantial commitments exist, there are notable gaps and overlaps in definitions, implementation, and outcome measurements. First, many documents address accessibility and support for individuals with disabilities, yet they often employ varying definitions and frameworks. For example, while the “Rights of Persons with Disabilities Act” clearly outlines specific legal protections and mandates for digital accessibility across sectors,^53^ other documents like the Digital Government Policies heavily emphasize compliance with international standards without a clear connection to how these standards address the unique needs of various disability groups.^56^ This inconsistency could lead to uneven implementation of policies meant to protect and empower individuals within vulnerable populations.

Further complicating the landscape are discrepancies in the framing of vulnerable groups themselves. Documents often conflate various marginalized identities, such as individuals with disabilities, the elderly, and those living in remote areas, under broad terminology without specifying distinct accessibility needs.^54^ For instance, the Digital Inclusion National Platform repeatedly mentions “vulnerable communities” without detailing how the unique barriers faced by each group are specifically addressed.^54^ In practice, this could result in policies that fail to consider, for example, the different types of support required for neurodiverse individuals versus elderly users, leading to one-size-fits-all solutions that do not effectively serve the intended populations.

Moreover, the implementation of digital literacy programs designed to assist these populations shows variability across different documents. While the Digital Inclusion Program emphasizes the importance of enhancing digital skills for individuals with disabilities through targeted training initiatives articulates robust training objectives, it does not offer concrete examples of how such programs are tailored to different populations, resulting in a lack of clarity on effectiveness,^52,56^ the specific types of training, metrics for success, and how they relate to educational outcomes remain ambiguous. Some documents suggest a commitment to training without providing measurable goals or outcomes, which could lead to inconsistent support experiences across various regions and sectors articulates robust training objectives, it does not offer concrete examples of how such programs are tailored to different populations, resulting in a lack of clarity on effectiveness.^52,59^ For example, while the Ministry of Human Resources and Social Development articulates robust training objectives, it does not offer concrete examples of how such programs are tailored to different populations, resulting in a lack of clarity on effectiveness.^52^

Lastly, despite the robust framework of digital access initiatives established by these policies, there is often a lack of follow-up or feedback mechanisms to assess their impact effectively. For example, while the systems are in place for improving access through assistive technologies and inclusive platforms,^60^ many documents fail to report on specific outcomes or user experiences, which creates uncertainty about the real-world effectiveness of these measures. The disconnect between outlined initiatives and their actual implementation may create a situation where vulnerable groups do not receive the comprehensive support they require. Without established accountability measures or transparent reporting practices, it becomes increasingly challenging to ensure that policies deliver genuine benefits to those they are intended to help, highlighting a significant inconsistency in the overall approach to digital inclusion for vulnerable communities in Saudi Arabia.

### Q5. What Temporal Details Describe Developments in Policies That Affect Digital Inclusion for These Groups?

To understand the temporal developments in policies affecting digital inclusion for vulnerable groups in Saudi Arabia, it is essential to examine the chronological progression of key legislative and programmatic milestones. These include foundational international commitments, regional agreements, and specific domestic laws, all contributing to the landscape of digital accessibility. The evolution of these policies is framed significantly by the broader Vision 2030 initiative, which acts as a guiding mechanism toward inclusive digital governance and the empowerment of marginalized populations. The “Rights of Persons with Disabilities Act,” enacted in 2019, represents a pivotal legal anchor within this framework, mandating comprehensive accessibility across public and private sectors.^53^

Significant temporal markers also highlight crucial development phases for digital inclusion initiatives. For instance, the national effort to connect over 3000 remote schools through satellite technology emerged as a key project in 2020, demonstrating a commitment to bridging geographic gaps in accessibility.^54^ Additionally, the launch of the Digital Inclusivity Program by the Digital Government Authority on November 22, 2023, exemplifies a focused implementation phase as part of ongoing digital transformation efforts.^56^ Such timelines indicate that Saudi Arabia is actively pursuing strategic objectives aimed at enhancing digital access while ensuring that the needs of vulnerable populations are met through dedicated operational plans.

Moreover, the overarching policy framework includes structured review and modification processes to ensure the continuous evolution of accessibility standards. The Accessibility Policy from the Ministry of Human Resources and Social Development articulates a commitment to annual reviews of digital services to maintain compliance with local regulations and address emerging technological advancements.^52^ Additionally, the Introductory Guide Digital Inclusion Program outlines plans for regular policy assessments and the implementation of maturity indices to evaluate progress toward digital inclusivity.^56^ This combination of strategic long-term objectives and responsive review processes underscores a proactive approach to ensuring that digital inclusion remains relevant and effective in meeting the needs of all citizens, particularly those in vulnerable or marginalized positions.

### Q6. What Outcomes Are Linked to Initiatives Targeting Digital Access in Health or Education for Neurodiverse or Disabled Populations?

In examining the outcomes linked to initiatives targeting digital access in health and education for neurodiverse and disabled populations in Saudi Arabia, it becomes apparent that various programs and strategies have led to measurable improvements. For instance, digital health initiatives have made significant strides, as evidenced by a video call center service that offers sign language interpretation for emergency assistance, reaching over 35 000 individuals with hearing impairments.^58^ Furthermore, the use of hospital communication applications has been shown to reduce anxiety for 37% of patients, indicating that digital health services are not only expanding access but also enhancing the quality of care for disabled populations. These outcomes signify a tangible impact on health service delivery, showcasing how targeted digital access strategies can translate into improved healthcare experiences for users with disabilities.

In the educational sector, initiatives such as the Madrasati digital platform have garnered international recognition for their effectiveness in accommodating students with disabilities, particularly during the COVID-19 pandemic. The platform enabled various electronic devices to support learning, which was acknowledged by UNESCO as one of the top 4 global models for distance learning during this critical period.^56^ Moreover, the integration of features like real-time captioning and text-to-speech within educational systems exemplifies specific efforts to ensure that students with disabilities are able to engage fully in virtual classrooms.^53^ While the evidence of outcomes primarily highlights successes in health, the educational measures underscore the broader commitment to inclusivity in Saudi Arabia’s digital initiatives, reaffirming that when accessible technologies are effectively implemented, they can significantly benefit neurodiverse and disabled populations.

### Q7. What Barriers or Challenges Are Identified for Individuals With Disabilities, Neurodiverse Users, or Other Vulnerable Communities?

In examining the barriers and challenges faced by individuals with disabilities, neurodiverse users, and other vulnerable communities in Saudi Arabia, several critical issues emerge from the policy documents. A significant barrier identified is the physical inaccessibility to government services, particularly in traditional settings. For instance, many individuals with disabilities encounter substantial mobility limitations that prevent them from attending notarial offices or civil status offices.^59^ The policy documents outline these challenges clearly, noting that special services are required for those who cannot easily access physical locations, including elderly individuals and patients.^54^ This physical barrier highlights the need for remote and alternative service delivery models to ensure that all citizens, regardless of their physical capabilities, can obtain essential documentation and services.

Another key challenge reported in the documents pertains to digital literacy deficits that hinder effective participation in the digital society for vulnerable groups. The Digital Inclusion National Platform explicitly notes the prevalence of “digital illiteracy,” particularly among elderly individuals and persons with disabilities, emphasizing the need for comprehensive training initiatives to equip these populations with necessary digital skills Overall, the recognition of these challenges serves as a critical first step toward developing targeted interventions but suggests that further refinement in approach and execution is necessary to achieve meaningful digital inclusion for all vulnerable communities.^54^ Furthermore, documents indicate that as individuals age or acquire disabilities, their ability to navigate digital tools may decrease, which can lead to increased risks of social isolation Overall, the recognition of these challenges serves as a critical first step toward developing targeted interventions but suggests that further refinement in approach and execution is necessary to achieve meaningful digital inclusion for all vulnerable communities.^56^ While acknowledging these barriers is crucial, the lack of specific metrics or outcomes tied to skill-building initiatives raises concerns about the effectiveness and reach of proposed solutions. Overall, the recognition of these challenges serves as a critical first step toward developing targeted interventions but suggests that further refinement in approach and execution is necessary to achieve meaningful digital inclusion for all vulnerable communities.

### Q8. What Recommended Actions or Planned Interventions Aim to Improve Digital Inclusion for These Populations?

To enhance digital inclusion for vulnerable populations, including those with disabilities and neurodiverse individuals, various comprehensive actions and planned interventions have been outlined in Saudi Arabia’s policy documents. One significant recommendation is the implementation of international accessibility standards, particularly the Web Content Accessibility Guidelines (WCAG). The Accessibility Policy from the Ministry of Human Resources and Social Development mandates adherence to these standards, requiring that all digital services comply with WCAG 2.1 Level AAA to ensure accessibility for all individuals.^52^ Additionally, the Digital Inclusion National Platform emphasizes the modernization of government websites and application interfaces to incorporate accessibility features such as high-contrast modes, text resizing options, and alternative texts for images, which collectively aim to create a user-friendly environment for all citizens.^54^

In addition to technical measures, substantial initiatives targeting digital literacy are critical for overcoming skills gaps identified among vulnerable populations. The Digital Inclusion Program emphasizes the development of comprehensive educational initiatives tailored specifically for individuals with limited digital skills, including elderly users and persons with disabilities.^56^ Notably, these programs aim to equip users with essential digital competencies through specialized training sessions and workshops, ensuring their effective participation in the digital landscape.^59^ Furthermore, the integration of assistive technologies into educational platforms and services is highlighted as a crucial intervention, with the 2019 Disability Law mandating the incorporation of features such as real-time captioning and text-to-speech functionalities.^53^ Together, these planned actions demonstrate a clear commitment to creating an inclusive digital environment that not only meets accessibility standards but also empowers vulnerable groups through skill development and targeted technological support.

### Q9. Which Groups or Stakeholders Are Referenced as Central to Achieving Equitable Access for Neurodiverse and Vulnerable Communities?

To identify the key groups and stakeholders central to achieving equitable access for neurodiverse and vulnerable communities in Saudi Arabia, it is essential to recognize the multi-faceted approach adopted by the government. Primary among these stakeholders are various governmental entities, notably the Authority of People with Disability (APD) and the Digital Government Authority (DGA). The APD is described as the “umbrella organization for all matters pertaining to persons with disabilities,” with a commitment to empowering this community and ensuring their rights are upheld in collaboration with other agencies.^59^ The DGA plays a significant role in managing the Digital Inclusivity Program and actively partners with the Ministry of Human Resources and Social Development to develop policies and frameworks that enhance digital access across the nation.^56^ Together, these entities form a crucial coalition aimed at improving inclusivity in public services, particularly for individuals who face barriers to accessing digital health and education resources.

In addition to government agencies, the involvement of persons with disabilities themselves is emphasized throughout the policy framework as essential stakeholders in the process of achieving equitable digital access. Their active participation in the design and evaluation of services is consistently highlighted, ensuring that the solutions developed are responsive to their actual needs.^54^ Reports detail the necessity for direct engagement with users to inform policy decisions and service improvements, with the DGA articulating that “Electronic Participation” will be a key pillar of their strategy.^56^ Furthermore, the integration of private sector partners and civil society organizations is also recognized as vital. The Digital Government Policies document mandates collaboration among government agencies, private stakeholders, and civil groups to facilitate a more inclusive digital environment.^56^ This tripartite collaborative approach reflects a holistic understanding that achieving digital equity requires a concerted effort from multiple sectors, encompassing not just the design of digital services but also the infrastructure and community support necessary for effective implementation.

### Q10. What Language Choices or Framing Strategies Are Used When Discussing Disability, Neurodiversity, or Vulnerable Groups in the Context of Digital Inclusion?

When discussing disability, neurodiversity, or vulnerable groups in the context of digital inclusion, the language choices and framing strategies employed in Saudi Arabia’s policy documents reflect a conscious effort to uphold dignity and promote empowerment. Predominantly, the documents utilize person-first language, which emphasizes the individuality of those affected rather than defining them solely by their disabilities. For instance, the Accessibility Policy mentions “people with disabilities and the elderly,” positioning these individuals before their conditions.^52^ This approach aligns with international best practices in disability discourse, reinforcing the idea that individuals should not be labeled or reduced to their disabilities. Furthermore, terms like “accessibility” are framed as universal design principles, highlighting their importance for all users rather than as specialized accommodations for select groups. This language choice indicates a commitment to inclusivity that benefits the broader society, suggesting that accessibility is a priority for good service delivery rather than an afterthought.

Despite these progressive elements, there are notable inconsistencies and gaps in how neurodiversity is articulated within the documents. Many policies subsume neurodiversity under general disability categories or rely on medicalized language that does not recognize neurodiversity as a distinct aspect of human cognition. For example, while some documents reference neurodiverse populations, they often do so through terms like “people with autism disorder,” which lacks the affirming language typically associated with a neurodiversity framework.^58^ Such framing may inadvertently reinforce outdated perceptions that view neurodiversity primarily as a deficit requiring special treatment. The repetition of vulnerability-based language, where persons with disabilities are grouped with other marginalized communities, further complicates the narrative by blending distinct needs into a generalized framework of support.^54^ This may dilute the specific rights and requirements pertinent to individuals with disabilities and neurodiverse conditions, potentially leading to policies that fail to fully accommodate or recognize the unique experiences and capabilities of these populations.

**Figure 2.**
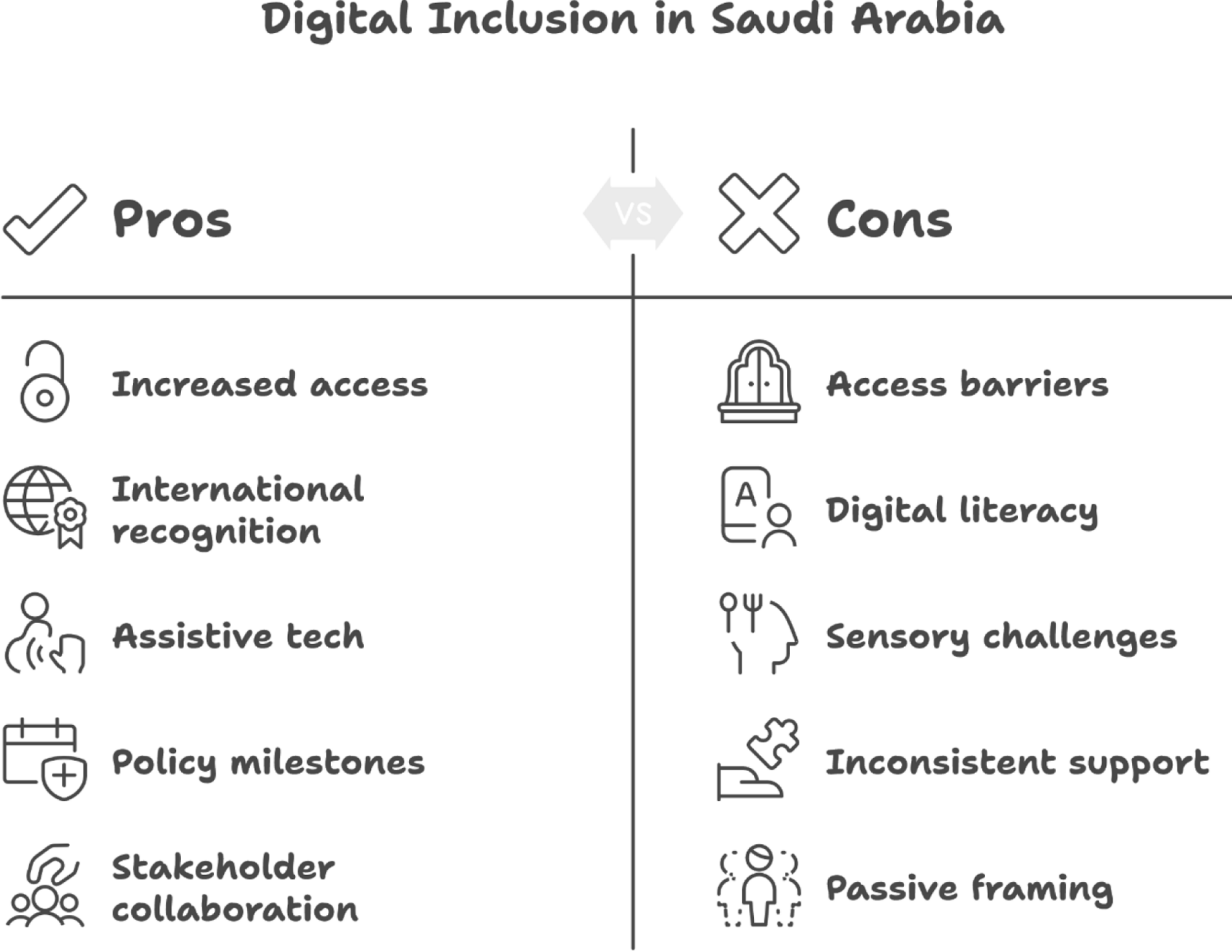
Pros and cons of current digital inclusion system in Saudi Arabia based on the analyzed documents.

Figure 2 illustrates a comparative overview of the strengths and limitations shaping digital inclusion efforts in Saudi Arabia. The pros highlight areas of progress, including expanded access to digital services, growing international recognition, the adoption of assistive technologies, key policy advancements, and active collaboration among stakeholders. In contrast, the cons emphasize ongoing challenges such as physical and digital access barriers, limited digital literacy among vulnerable groups, unmet sensory-related accessibility needs, inconsistent levels of support across sectors, and the persistence of passive or deficit-oriented framing in policy language.

### Digital Inclusion and Sustainable Development Goals

#### Sustainable Development Goal 3 (SDG 3)

*Aim: To ensure healthy lives and promote well-being for all at all ages.*

Saudi Arabia’s initiatives directly support SDG 3 by enhancing digital health access for vulnerable populations. For example, emergency services utilizing video call centers for sign language interpretation have benefited over 35 000 hearing-impaired individuals, thereby improving the quality of healthcare participation for this group.^54^ Additionally, studies indicate that hospital communication applications have alleviated anxiety for 37% of patients, highlighting the psychological benefits of accessible healthcare services.^54^ These efforts align with the overarching goal of ensuring that all demographic groups can access the health services they need, ultimately contributing to a more inclusive healthcare environment across Saudi Arabia.

#### Sustainable Development Goal 4 (SDG 4)

*Aim: To ensure inclusive and equitable quality education and promote lifelong learning opportunities for all.*

The documents analyzed reveal a commitment to enhancing digital educational access, crucial for achieving SDG 4. The Madrasati platform, recognized globally during the COVID-19 pandemic, served as a vital educational resource for students with disabilities, providing them with necessary technological support.^56^ Furthermore, policy frameworks emphasize the inclusion of adaptive learning tools, real-time captioning, and tailored learning environments to ensure that students with disabilities can fully engage in virtual classrooms.^53^ This concerted effort to provide equitable educational opportunities reflects Saudi Arabia’s commitment to eliminating disparities in access to education for all citizens.

#### Sustainable Development Goal 10 (SDG 10)

*Aim: To reduce inequalities based on age, sex, disability, race, ethnicity, origin, religion, or economic status.*

In addressing SDG 10, the analyzed documents demonstrate that Saudi Arabia recognizes the need to empower vulnerable communities, particularly through participatory methodologies. The Authority of People with Disability has articulated its focus on including the voices of persons with disabilities in shaping social support programs, thereby fostering a more inclusive society.^59^ Additionally, collaboration is emphasized as a critical component in achieving equitable access, requiring the concerted efforts of government agencies, private sectors, and civil society organizations to promote equality and fairness in service delivery.^56^ These initiatives are essential for ensuring that all citizens, especially those from marginalized backgrounds, can fully participate in society.

#### Target 3.8 and Target 4.5

*Aim of 3.8: To achieve universal health coverage, including financial risk protection and access to quality essential healthcare services. Aim of 4.5: To eliminate disparities in education and ensure equal access for all levels of education and vocational training for vulnerable populations, including persons with disabilities.*

The documents collectively highlight the importance of access to quality health and educational services, particularly for vulnerable populations. The Accessibility Policy from the Ministry of Human Resources and Social Development mandates compliance with international accessibility standards, targeting essential service coverage across healthcare and education to ensure inclusivity.^52^ Additionally, the focus on developing comprehensive training programs for both end-users and service providers indicates a strategic commitment to build capacity that aligns with the aim of achieving universal health coverage and equitable access to education.^56^

#### Indicators 3.8.1 and 4.5.1

*Indicator 3.8.1: Coverage of essential health services (based on tracer interventions). Indicator 4.5.1: Parity indices for education indicators disaggregated by various demographic factors.*

While not explicitly linked to these indicators, the examined documents suggest a foundational capability to gauge progress in health and education. For instance, targeted health initiatives that enhance service coverage for persons with disabilities provide a basis for tracking essential health services.^54^ Furthermore, the success of the Madrasati platform demonstrates potential pathways for assessing educational participation rates and addressing disparities that might arise based on disability status or educational access.^56^ These reflections underscore the connection between Saudi Arabia’s digital inclusion initiatives and their broader alignment with the SDGs, emphasizing a commitment to inclusive growth that bridges gaps for vulnerable communities.

**Figure 3.**
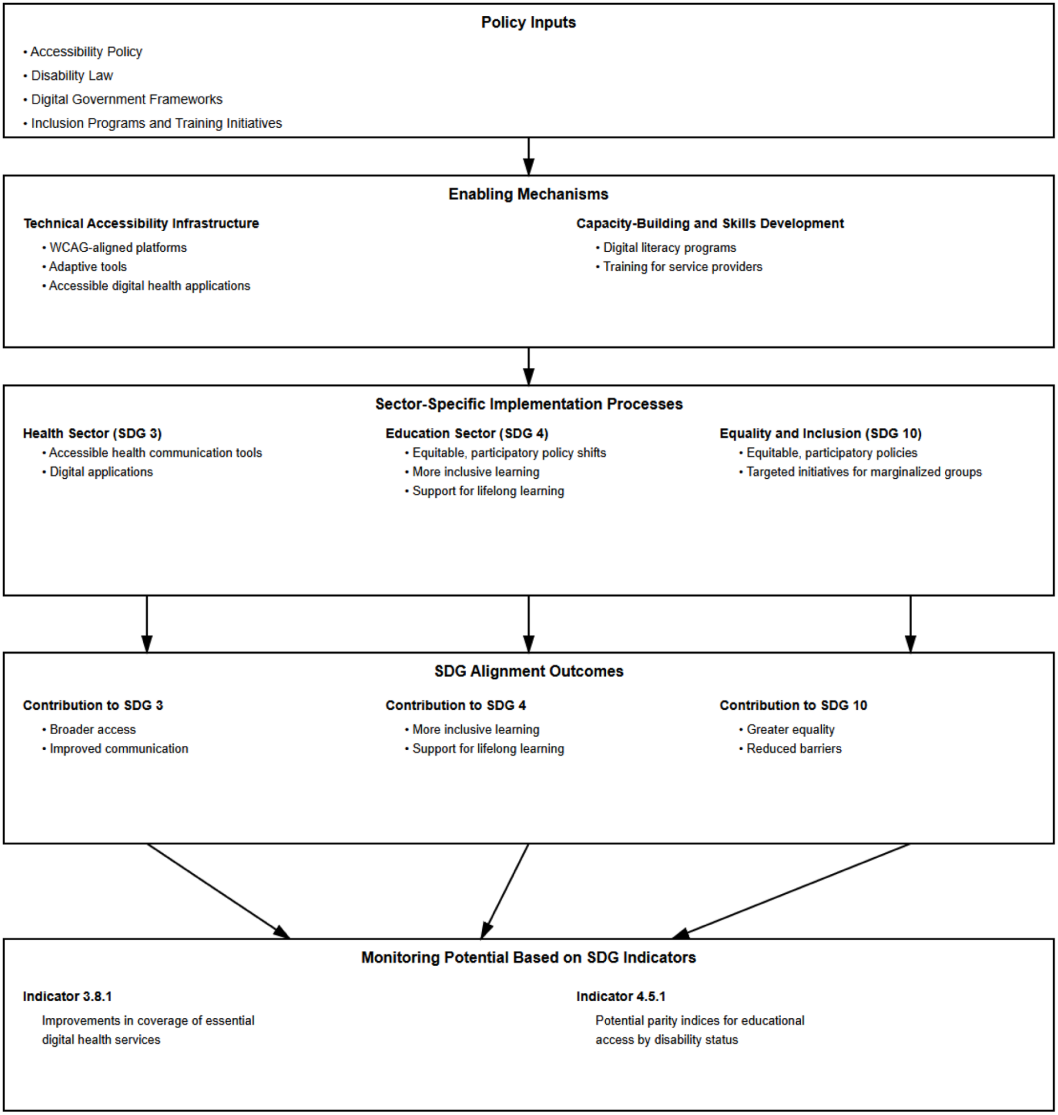
Conceptual framework of digital inclusion pathways supporting SDGs in Saudi Arabia.

Figure 3 presents a structured framework that demonstrates how Saudi Arabia’s digital inclusion policies translate into measurable contributions to key SDGs. The model begins with foundational policy inputs, such as accessibility regulations and disability rights legislation, which establish the national commitment to equitable digital access. These inputs activate enabling mechanisms that include both technical accessibility infrastructure and capacity-building initiatives designed to enhance digital skills among vulnerable populations. Through these mechanisms, sector-specific processes in health, education, and inclusion are implemented, resulting in improved access to essential services, more inclusive learning environments, and reductions in structural inequalities. The framework also highlights the emerging potential for monitoring progress through SDG-relevant indicators, illustrating a policy ecosystem that is increasingly capable of assessing and sustaining advancements in digital equity.

Taken together, the 10 analytical questions addressed above correspond to the 3 aims of the study. Aim 1—examining how digital inclusion is conceptualized and framed—is addressed through Q1 (definitions and descriptions), Q3 (assumptions about digital needs and abilities), and Q10 (language and framing strategies). Aim 2—identifying evidence, outcomes, and challenges related to digital access in health and education—is addressed through Q2 (types of evidence), Q5 (temporal policy developments), Q6 (outcomes of initiatives), and Q7 (barriers and challenges). Aim 3—analyzing proposed actions, stakeholders, and strategies—is addressed through Q4 (inconsistencies in support), Q8 (recommended actions and interventions), and Q9 (stakeholder involvement). This mapping confirms that the analytical framework thoroughly addresses all 3 research aims and provides a structured basis for the discussion that follows.

## Discussion

The present study was motivated by the observation that healthcare and education in Saudi Arabia often function as disconnected systems, creating fragmented services that disrupt support for people with disabilities and neurodevelopmental differences. Consequently, this research aimed to examine how national policy documents conceptualize digital inclusion, identify reported evidence and challenges, and analyze proposed strategies for equitable participation. The results indicated that evidence of digital access primarily relies on international benchmarks and demographic data. Underlying assumptions frequently conflate disability with digital illiteracy, prioritizing technical conformity over user-centered design. Support mechanisms displayed inconsistencies, characterized by divergent definitions of vulnerability and uneven implementation. Policy developments, notably the 2019 Disability Law, mark a clear progression toward structured inclusion. Reported outcomes show gains in health and assistive technology adoption, though these remain restricted by contextual constraints. Barriers persist in the form of physical, sensory, and cognitive limitations. Recommended actions center on standardization and digital literacy. Stakeholder involvement is framed as a collaborative effort involving government, civil society, and individuals, while language patterns often employ passive or deficit-oriented framing. Finally, the SDG analysis revealed that while national strategies align with Goals 3, 4, and 10, these connections are often implicit rather than directly tracked against specific indicators.

The finding that policy documents tend to prioritize technical compliance and “conformity” over user experience highlights a critical gap in the current conceptualization of digital inclusion. Previous scholarship defines digital inclusion as a multidimensional construct that must transcend basic access to encompass skills, meaningful participation, and support systems.^1,6^ By focusing heavily on adherence to standards like WCAG, current frameworks may neglect the ecosystem of interconnected factors required for genuine participation. This aligns with arguments that true inclusion requires moving beyond a “usage layer” to a “skills layer” where individuals can effectively utilize platforms for self-sufficiency.^7^ Furthermore, the prevalence of deficit-oriented language in the analyzed documents contradicts emerging perspectives that advocate for recognizing the unique strengths of neurodivergent individuals to reduce bias and foster supportive environments.^61^

While the study identified a strong legislative foundation driven by Vision 2030, the results also pointed to significant inconsistencies in how support is defined and delivered across sectors. This mirrors broader regional challenges where excellent policy intent often encounters implementation hurdles due to limited provider competency or infrastructural gaps type of tailored support that recent studies identify as essential for psychological safety and workplace inclusion.^38^ Without precise definitions of vulnerability, services risk becoming fragmented, failing to address the intersectional nature of digital disadvantage.^5,37^ The literature suggests that disability-specific policies are generally more effective than broad diversity frameworks because they explicitly mandate accommodations and rights. The lack of explicit neurodiversity references in the analyzed texts suggests a missed opportunity to institutionalize the type of tailored support that recent studies identify as essential for psychological safety and workplace inclusion.^38^ Without precise definitions of vulnerability, services risk becoming fragmented, failing to address the intersectional nature of digital disadvantage.^5^

The persistence of physical, sensory, and cognitive barriers identified in the results underscores that infrastructure alone cannot resolve digital exclusion. Research emphasizes that digital inequality often mirrors pre-existing social and economic disparities, meaning that groups facing geographic isolation or literacy challenges require more than just device distribution.^4^ The study’s finding that recommended actions focus on digital literacy is consistent with global calls to equip users with the competencies needed to navigate an evolving digital society.^3^ However, for these interventions to be effective, they must be adaptive and continuous, treating digital inclusion as a dynamic process rather than a one-time achievement. The reliance on standardization found in the documents may prove insufficient if it does not account for the rapid emergence of new technologies, such as the Metaverse, which bring novel accessibility and privacy concerns.

Finally, the analysis of SDGs suggests that Saudi Arabia’s digital strategies contribute to health (SDG 3), education (SDG 4), and reduced inequalities (SDG 10), yet often lack the explicit indicator tracking necessary for rigorous impact assessment. This implicit alignment limits the ability to measure progress comparable to international standards. Literature indicates that digital inclusion is a powerful driver for sustainable living and health outcomes, particularly for vulnerable populations like the elderly.^7,12^ To fully realize these benefits, governance frameworks must move beyond general alignment to embed fairness and human rights principles directly into the lifecycle of digital systems. Establishing concrete feedback loops and specific metrics for neurodiverse inclusion would allow for more precise monitoring of how digital transformation supports the broader national agenda of social equity.^2^ In short, while the Saudi context is shaped by specific national policies such as Vision 2030 and the 2019 Disability Law, the patterns identified—including the gap between legislative intent and implementation, reliance on technical compliance over user-centered design, and the conflation of disability with digital illiteracy—are broadly representative of challenges facing Global South nations navigating digital transformation under conditions of uneven infrastructure, limited capacity, and historically marginalized disability communities.^1,19^

### Recommendations

The findings point to several opportunities for strengthening digital inclusion policy and practice in Saudi Arabia. First, policies should move beyond technical accessibility compliance to prioritize user-centered design that reflects the lived experiences of neurodiverse individuals and other vulnerable groups, as highlighted in recent work showing that fragmented systems fail to meet the needs of learners and service users across sectors cultivate a more coordinated and inclusive digital ecosystem that supports health and learning outcomes for vulnerable groups.^43,46^ Second, clearer and more consistent definitions of disability, vulnerability, and neurodiversity are needed to avoid fragmented implementation and to ensure that policies respond to the specific needs of diverse groups. This should be paired with structured participatory mechanisms that position people with disabilities as active contributors to policy development and system design. Third, investment in capacity-building is essential, including targeted digital literacy programs for users and specialized training for providers across healthcare and education. Finally, monitoring systems aligned with SDG indicators—such as service coverage, parity indices, and equitable access metrics—should be institutionalized to track progress and support adaptive policy refinement. These steps would help cultivate a more coordinated and inclusive digital ecosystem that supports health and learning outcomes for vulnerable groups.

### Limitations

This study is limited by its reliance on 9 policy and grey documents, which offer official perspectives but do not fully capture lived experiences or the practical realities faced by neurodiverse individuals and people with disabilities in Saudi Arabia. Prior research similarly notes mismatches between policy intentions and implementation, particularly due to siloed service structures and inconsistent cross-sector coordination.^43,46^ Document analysis cannot determine whether policies are applied uniformly across regions or institutions, nor can it capture user experiences, staff practices, or informal barriers such as stigma, digital literacy gaps, or variable institutional capacity. The analysis is further constrained by inconsistencies in terminology and reporting across documents and by uneven levels of detail in policy descriptions, which may influence interpretation. Finally, the study focuses on national-level documents and does not examine regional or institutional strategies, which may offer additional nuance. These limitations point to the need for future empirical work involving policymakers, service providers, families, and disabled users to complement document-based insights and evaluate the real-world impact of digital inclusion policies.^62^

## Conclusion

This study examined digital inclusion for neurodiverse and vulnerable populations in Saudi Arabia, recognizing that healthcare and education systems often operate as disconnected entities, resulting in fragmented services and limited continuity of support for individuals with disabilities or neurodevelopmental differences. Through Framework Analysis of 9 national policy and grey documents, the research aimed to understand how digital inclusion is conceptualized, identify evidence and challenges related to digital access in health and education, and analyze proposed strategies for advancing equitable participation. The analysis revealed that while Saudi Arabia demonstrates strong legislative commitment through the 2019 Disability Law and Vision 2030 initiatives, digital inclusion efforts rely heavily on international accessibility benchmarks and technical compliance rather than user-centered design. The findings indicated persistent gaps including the conflation of disability with digital illiteracy, inconsistent support mechanisms across sectors, and deficit-oriented language framing. Despite reported improvements in health service delivery—such as sign language interpretation reaching over 35 000 individuals and educational platforms like Madrasati gaining international recognition—barriers related to physical accessibility, digital literacy deficits, and uneven implementation continue to limit meaningful participation. Although national strategies align with SDGs 3, 4, and 10, this alignment remains largely implicit without systematic indicator tracking, highlighting the need for more explicit monitoring systems, participatory design approaches, and coordinated cross-sector investment to realize truly inclusive digital ecosystems that support both health and educational outcomes for vulnerable communities.

## Supplemental Material

sj-docx-1-inq-10.1177_00469580261443129 – Supplemental material for Digital Inclusion for Neurodiverse and Vulnerable Communities in the Global South: A Policy Analysis Study Supplemental material, sj-docx-1-inq-10.1177_00469580261443129 for Digital Inclusion for Neurodiverse and Vulnerable Communities in the Global South: A Policy Analysis Study by Ahmed Yahya Almakrob, Ahmed Alduais, Alex Mhone, Borey Be, Tahira Yasmeen and Tariq Alanazi in INQUIRY: The Journal of Health Care Organization, Provision, and Financing
