# Supplementary material for: Digital Inclusion for Neurodiverse and Vulnerable Communities in the Global South: A Policy Analysis Study
Source: Inquiry. 2026 Apr 24;63:00469580261443129. doi: 10.1177/00469580261443129 (PMC13125792; doi:10.1177/00469580261443129)
Supplement: sj-docx-1-inq-10.1177_00469580261443129 – Supplemental material for Digital Inclusion for Neurodiverse and Vulnerable Communities in the Global South: A Policy Analysis Study [file sj-docx-1-inq-10.1177_00469580261443129.docx]

**SRQR Reporting Checklist – Completed**

*Manuscript: Digital Inclusion for Neurodiverse and Vulnerable Communities in the Global South: A Policy Analysis Study*

Manuscript ID: INQ-25-0967 | Reporting Guideline: SRQR (O'Brien et al., 2014)

| **Item** | **Description** | **Location in Manuscript** |
| --- | --- | --- |
| **Title & Abstract** | | |
| **Title** | Describe the nature and topic of the study. Identify the study as qualitative or indicate the approach or data collection methods. | *Title page. The title explicitly states 'A Policy Analysis Study', identifying the qualitative approach and the topic (digital inclusion for neurodiverse and vulnerable communities in the Global South).* |
| **Abstract** | Summarise the key elements of the study using the abstract format of the intended publication. | *Abstract (p. 1). The abstract summarises the background, aims, method (Framework Analysis of 9 policy documents), results (10 thematic areas), and conclusions, including SDG alignment.* |
| **Introduction** | | |
| **Problem Formulation** | Describe the problem/phenomenon studied, its significance, relevant theory and empirical work, and gaps in current knowledge. | *Introduction, pp. 1–5. Three sub-sections establish the conceptual foundations of digital inclusion, review international policy initiatives, and describe the Saudi Arabian context, culminating in an explicit gap statement immediately before 'The Present Study'.* |
| **Purpose or Research Question** | Describe the purpose of the study and specific objectives or questions. | *The Present Study section, p. 5. Three study aims are stated: (1) how digital inclusion is conceptualised in policy documents; (2) evidence, outcomes and challenges in digital health and education; (3) proposed actions, stakeholders and strategies for equitable participation.* |
| **Methods** | | |
| **Qualitative Approach and Research Paradigm** | Describe the qualitative approach, guiding theory (if appropriate), research paradigm, and reasons for choices. | *Methods – Design, p. 5. Framework Analysis (Ritchie & Spencer, 2002; Gale et al., 2013) is identified as the methodological approach. Its suitability for health, education and social policy research, and its capacity to integrate inductive and deductive perspectives while maintaining transparency, are explicitly justified.* |
| **Researcher Characteristics and Reflexivity** | Describe how researchers' characteristics may influence the research, including qualifications/experience, assumptions, and presuppositions; potential interaction between characteristics and research questions, methods, results and/or transferability. | *Methods – Measures, p. 6. Reflexivity is addressed: the study acknowledges the researcher's positionality, professional background, and potential influence on interpretation as part of the trustworthiness strategy. All six authors participated in the document review to reduce individual interpretive bias.* |
| **Context** | Describe the setting/site(s) in which the study was conducted, why it was selected, and any other salient contextual factors. | *Introduction – Digital Inclusion for People with Disabilities in Saudi Arabia, pp. 3–5; and The Present Study, p. 5. Saudi Arabia is situated as a Global South context shaped by Vision 2030 and the 2019 Disability Law, with healthcare and education operating as disconnected systems.* |
| **Sampling Strategy** | Describe how and why research participants, documents, or events were selected; criteria for deciding when no further sampling was necessary, and the rationale for those criteria. | *Methods – Sample, pp. 5–6 and Table 1, p. 6. Purposive sampling was used to select nine documents directly relevant to digital inclusion in Saudi Arabia. The rationale for inclusion criteria (national strategies, regulatory frameworks, accessibility standards) and the diversity of governmental sources are described. Documents were publicly available online via official Saudi governmental portals.* |
| **Ethical Issues Pertaining to Human Subjects** | Describe any approval by an appropriate ethics review board and participant consent, or explain any lack thereof. Describe any other confidentiality and data security issues. | *Declarations section, p. 22. The study did not involve human or non-human subjects; therefore, IRB approval and informed consent were not required. No confidentiality issues arise as all nine documents are publicly available.* |
| **Data Collection Methods** | Describe the types of data collected; details of data collection procedures including start/stop dates, iterative process, triangulation of sources/methods, and modification of procedures in response to evolving study findings. | *Methods – Sample, pp. 5–6; Methods – Procedure, pp. 6–7. Data consisted of nine publicly available policy and grey documents retrieved from official Saudi governmental websites. Triangulation was achieved by drawing on documents from multiple governmental bodies. The analytic process followed five iterative stages: familiarisation, framework development, indexing, charting, and interpretation.* |
| **Data Collection Instruments and Technologies** | Describe any instruments (e.g., interview guides, questionnaires) and devices used for data collection; describe if/how instruments changed over the course of the study. | *Methods – Procedure, pp. 6–7. The Framework Analysis matrix served as the primary data extraction and organisation instrument, structured around 10 analytic questions aligned with the study aims. No audio or digital recording devices were required given the documentary nature of the data.* |
| **Units of Study** | Describe the number and relevant characteristics of participants, documents, or events included in the study. Describe the level of participation. | *Methods – Sample, pp. 5–6 and Table 1, p. 6. Nine policy and grey documents were included, representing a range of Saudi governmental bodies: Ministry of Human Resources and Social Development, Digital Government Authority, Authority of People with Disability, and Saudi Data and Artificial Intelligence Authority. Document types include accessibility policies, disability legislation, digital government guidelines, strategic frameworks, and programmatic reports.* |
| **Data Processing** | Describe the methods for processing data prior to and during analysis, including data entry, data management, verification of data integrity, data coding, and anonymisation/de-identification. | *Methods – Procedure, pp. 6–7. Each document was systematically read and re-read by the research team (all six authors). Significant passages were highlighted and recurring themes identified. Data were charted into a structured matrix organised by analytic question and document source. No anonymisation was required as all documents are publicly available official sources.* |
| **Data Analysis** | Describe the process by which inferences, themes, etc. were identified and developed, including the researchers involved in data analysis; usually references a specific paradigm or approach. Describe why this process was chosen. | *Methods – Procedure, pp. 6–7; Results, p. 8. Framework Analysis (Ritchie & Spencer, 2002) was applied through five stages: familiarisation, thematic framework development, indexing, charting, and interpretation/mapping. Ten thematic areas were derived through the indexing and charting stages. All six authors participated. The method was selected for its structured, transparent, and audit-ready approach suited to multi-document policy analysis.* |
| **Techniques to Enhance Trustworthiness** | Describe any techniques to enhance trustworthiness and credibility of data analysis (e.g., member checking, triangulation, audit trail). Describe why these techniques were chosen. | *Methods – Measures, pp. 5–6. Four trustworthiness strategies are described: (1) credibility through prolonged engagement and triangulation across multiple governmental document sources (Bowen, 2009); (2) dependability through the systematic, replicable stages of Framework Analysis (Gale et al., 2013); (3) confirmability through an audit-like trail of coding decisions and analytic memos (Lincoln & Guba, 1985); (4) transferability through detailed contextual descriptions and diverse policy sources. Reflexivity was maintained throughout.* |
| **Results** | | |
| **Synthesis and Interpretation** | Describe the main findings (e.g., interpretations, inferences, and themes); might include development of a theory or model, or integration with prior research or theory. | *Results, pp. 8–19. Ten thematic areas are presented (Figure 1): digital access evidence, assumptions about needs, inconsistencies in support, temporal policy developments, outcomes of initiatives, barriers and challenges, recommended actions, stakeholder involvement, language and framing, and concluding observations. A summary paragraph at the end of Results maps Q1–Q10 to the three study aims. Figures 2 and 3 synthesise findings visually.* |
| **Links to Empirical Data** | Provide evidence (e.g., quotes, field notes, text excerpts, photographs) to substantiate analytic findings. | *Results, pp. 8–19 (Q1–Q10). Each thematic section is substantiated by direct citations to specific passages and language from the nine policy documents (e.g., Ministry of Human Resources and Social Development, 2025; Digital Government Authority, 2023; 2025; Saudi Data and Artificial Intelligence Authority, 2025a; 2025b; Authority of People with Disability, n.d.; Skynet Technologies, 2025). Direct textual examples from policy documents are referenced throughout.* |
| **Discussion** | | |
| **Integration with Prior Work, Implications, Transferability, and Contribution(s) to the Field** | Summarise the main findings, explain how findings and conclusions connect to, support, elaborate on, or challenge earlier scholarship; discuss scope of application/generalizability; identify unique contribution(s). | *Discussion, pp. 20–22. Findings are integrated with prior literature on digital inclusion (Jamil, 2021; Liao et al., 2022; Yan & Xing, 2025), neurodiversity policy (Putnam et al., 2025; Zaino, 2025), and Global South digital governance (Győrffy et al., 2023). The Saudi case is positioned as broadly representative of Global South nations facing similar policy-implementation tensions. Transferability is discussed explicitly.* |
| **Limitations** | Discuss the trustworthiness and limitations of findings. | *Limitations, p. 22. Limitations include: reliance on official policy documents (not lived experience data); inability to verify uniform implementation across regions; inconsistencies in terminology across documents; focus on national rather than regional/institutional strategies; and the absence of perspectives from service providers, families, and disabled users. Future empirical work is recommended.* |
| **Other** | | |
| **Conflicts of Interest** | Describe any potential sources of influence or perceived influence on study conduct and conclusions. Describe how these were managed. | *Declarations – Conflict of Interest, p. 23. The authors report no conflict of interest.* |
| **Funding** | Describe sources of funding and other support. Describe the role of funders in data collection, interpretation, and reporting. | *Declarations – Funding Statement, p. 22. Funding details are provided (removed for peer review in the submitted version; to be reinstated in the final manuscript).* |

**How this checklist was used**

The SRQR reporting guideline was used to guide manuscript preparation and editing. This completed checklist is submitted as a supplementary file. Items are reported in the manuscript body, tables, and figures as indicated above.

**Citation**

O'Brien, B. C., Harris, I. B., Beckman, T. J., Reed, D. A., & Cook, D. A. (2014). Standards for reporting qualitative research: a synthesis of recommendations. *Academic medicine : journal of the Association of American Medical Colleges*, *89*(9), 1245–1251. <https://doi.org/10.1097/ACM.0000000000000388>
